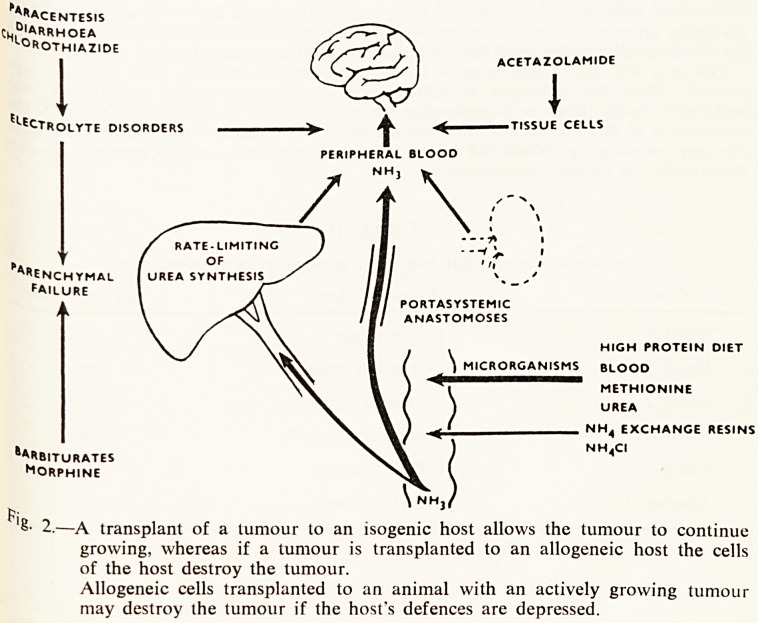# The Evolution of Liver Surgery
*An address to the Bristol Medico-Chirurgical Society, 9th March, 1966.


**Published:** 1966-07

**Authors:** A. G. Riddell

**Affiliations:** Professor of Surgery, University of Bristol


### THE EVOLUTION OF LIVER SURGERY*

BY

A. G. RIDDELL

*Professor of Surgery, University of Bristol*

*An address to the Bristol Medico-Chirurgical Society, 9th March 1966.

In all animals the liver is the largest single organ in the body and, not unnaturally, this has given rise to considerable speculation as to its function. In classical times the Greek warriors foretold the future by examining the variation in the hilar structures and the lobes of the livers from sacrificial animals. Little surgical interest, however, was displayed in the liver until the advent of vascular surgery, but following this there has been a rapid change.

The first significant scientific contribution to the study of the liver, both of its structure and function, was made by Glisson when in 1650 he published his famous book. We all remember Glisson for his capsule, but this is of little consequence compared with his discovery that the liver had a double blood supply, the hepatic artery and the portal vein. He wondered what the significance of this double blood supply was, and this observation was the real beginning of liver physiology. Glisson has another interest for you in that he was probably born in Bristol (Walker, 1966).

The elucidation of liver function did not appear until the mid-nineteenth century. The father of liver physiology is undoubtedly Claude Bernard, who discovered the presence of glycogen in the liver. Pavlov, not long after this, showed that the liver was the sole organ responsible for the synthesis of urea in the body; and around the turn of the century it was eventually proved that, although the liver excreted bile, bile pigment was formed in the reticulo-endothelial system throughout the body.

The fundamental discovery which led the way to the eventual growth of hepatic surgery was the discovery by von Podwyssozki in 1886 that, if he removed four-fifths of the liver of a rabbit, this rapidly regenerated. Much work has been done on this interesting phenomenon. For instance, regeneration of the rat liver is complete in 4 weeks, and correspondingly rapid and complete regeneration occurs in the liver of the dog and of man.

Let us recall that long ago an experiment was performed by an experimental pathologist called Zeus, working at Olympus, where he chained Prometheus to a rock, and each day he used an eagle to devour Prometheus's liver and each night Prometheus grew a new one. Somehow he managed to solve the problem of hæmostatis and the experiment is fundamentally sound, except that regeneration of liver in man is not quite at this rate; in fact regeneration of liver after massive resection is slow compared with that in lower animals. It takes about six weeks for regeneration to be complete in man and, during this period, there is considerable depression of liver function. The most striking finding is the depression of albumin synthesis and, unless the patient is supported with massive infusions of intravenous albumin, recovery will not take place (McDermott *et al.*, 1963).

### LIVER INJURY

In some ways the management of liver injuries presents comparatively few problems. For a long time the bulk of medical opinion was that any sort of internal hæmorrhage due to trauma was best treated conservatively, and even as late as 1948, Aird in his book suggested that the majority of liver injuries could be treated conservatively. This is now known to be quite wrong, and many liver injuries are fatal if an operation is not performed.

Much that has been done to pioneer the treatment of abdominal injuries by immediate operation we owe to Robert Cooke (Cooke and Southwood, 1964). The survival of a patient with a ruptured liver depends on early recognition of the lesion, followed by immediate laparotomy. Of 13 patients so treated only 2 died. Not only is it now believed that operative interference is essential in liver injuries, but the technique of the operation has changed. Most injuries can be treated by simple suture. Severe lacerations involving destruction of a lobe may require hepatic lobectomy rather than packing of the laceration with gauze rolls (which should never be resorted to). The need to perform a partial hepatectomy for liver injury led to the development of this operation.

### HEPATECTOMY

One of the formidable problems of surgery has been the removal of large lesions in the liver, involving massive resection of liver tissue. The major part of this problem has been the control of hæmorrhage. Recent anatomical studies of the liver have made it possible, following almost directly on Glisson's original description, to elucidate the blood supply of the liver and determine a lobularity of pattern based on the hepatic veins, and made it possible to perform either right or left lobectomy. Although many large tumours have been removed from the liver, these were removed with only small margins of normal liver tissue around them, because it was feared that the removal of large masses of liver tissue in man might lead to liver failure.

Some years ago I had the opportunity of removing the left lobe of a liver. The lobe weighed 200 g., and in it there was only a small malignant tumour. So in this patient we removed approximately 198 g. of normal liver. The patient's convalescence was completely smooth and there was virtually no change in his liver function tests in the immediate post-operative period (Riddell, 1952).

Since then larger and bigger liver resections have been performed, and it has now been shown that massive resections of normal liver tissue can be performed, and regeneration will take place, so long as adequate supportive post-operative care is given during the period of regeneration. The indications for hepatectomy are shown in Table I.

TABLE I

INDICATIONS FOR HEPATIC LOBECTOMY

Severe lacerations
Large abscesses
Parasitic disease
Tumours
Access to bile ducts

I do not intend to go into the details of the operation, but basically either the right or left lobe may be removed. The important anatomical landmarks are the three hepatic veins, the central one lying in a plane running from the gall-bladder fossa to the inferior vena cava. This plane is the line of dissection for the removal of either lobe after ligation of the appropriate branch of the portal vein, hepatic artery, and hepatic duct at the hilum. On the right side some veins entering the side of the inferior vena cava require ligation.

### PORTACAVAL ANASTOMOSIS

One of the great advances in surgery of the liver has sprung from our ability to reorganise its blood supply as a possible method of ameliorating the effects of disease. The classical example of this is portal hypertension, and here again, Bristol has been in the forefront of the solution of this problem. Bristol should be justly proud of a triumvirate of medical men who have done so much for liver disease; Glisson in earlier times, and recently Cooke on liver injuries and Milnes Walker on portal hypertension (Walker, 1957).

Obstruction to the flow of blood through the liver gives rise to an increased pressure in the portal vein, and this is followed by the development of collateral channels between the splanchnic and systemic circulations. The most significant of these are the

œsophageal varices and the consequences of these are that they may give rise to hæmorrhage which could endanger life.

The only satisfactory operation for portal hypertension is a shunt operation which produces adequate diversion of portal blood and lowers portal pressure, and causes the varices to collapse and eventually disappear. The most important and significant of these operations is the operation of portacaval anastomosis. The results of the operation in the prevention of bleeding are excellent, in that an adequately performed portacaval anastomosis virtually protects the patient from any further hæmorrhage from his varices.

However, when we perform a portacaval anastomosis we do disorganise the physiology of the liver, and we divert the portal blood away from the liver so that the liver can no longer perform its normal detoxicating function on the products of absorption; and also, if the total liver blood flow is substantially reduced as a result of portacaval anastomosis, a certain amount of atrophy in the liver probably occurs, and certainly little or no regeneration of liver tissue will take place in the future in response to injury.

So, in doing this operation we inevitably, in many cases, produce metabolic cripples. For 50 per cent of our patients this does not give rise to any difficulty, hence the good results of the operation (Riddell and Wilkinson, 1964).

The most significant complication of the operation is the development of portasystemic encephalopathy. For the first ten years after the introduction of portacaval anastomosis in the treatment of portal hypertension, disabilities were attributed to the pre-existing liver disease, and it was not until 1952 that striking proof of the danger of portacaval anastomisis was produced. McDermott and Adams (1954) described the development of encephalopathy in a patient who had a portacaval anastomosis in the absence of liver disease. The patient developed repeated episodes of stupor or coma following the administration of various nitrogenous substances. After extensive investigation of this patient they came to the conclusion that the neurological disturbance was due to ammonia intoxication. Although this is not necessarily the only cause of hepatic coma, the concept of nitrogenous intoxication has become an important one in the management of hepatic coma. A great deal of the work that has since been done in elucidating the mechanisms involved stems directly from the investigation of this one patient (Fig. 1).

### PORTACAVAL TRANSPOSITION

Very recently the possibility of altering the blood supply to the liver in other ways has come to the attention of the surgeon in the interesting condition of glycogen storage disease. There are many types of glycogen storage disease and only certain of these are suitable for operation. Some three years ago in the States, Starzl used portacaval transposition as an experimental procedure, and found that it lowered the glycogen content of the normal liver. As a result of this he performed the operation on a child with glycogen storage disease (Starzl *et al.*, 1965).

In the operation of portacaval transposition, both the portal vein and the inferior vena cava are divided and then anastomosed end to end, so that the blood returning from the gut passes directly into the inferior vena cava, while the blood returning *via* the inferior vena cava from the lower half of the body enters the liver by way of the portal vein. The main consequence of the operation is that the glucose in the portal blood passes directly into the systemic circulation so that it may be metabolised by the tissues, rather than held as glycogen in the liver where it cannot be broken down.

I performed this operation on a seven year old boy with Type I glycogen storage disease about a year ago (Riddell and Davies, 1966). The child is doing well, has grown 2 inches in height, has become vigorous, and his intelligence has markedly increased.

### THE FUTURE OF LIVER SURGERY

I should now like to turn from what I believe are the established accomplishments of liver surgery to some possibilities for the future. Probably the commonest form of liver disease which the surgeon encounters is the presence of secondary tumours within the liver, and in the management of this condition we are only at the beginning of what could in the future be a profitable era of exploration.

Before we can go on to attempt new methods of treatment of this condition, we need to know more about how to diagnose secondary tumours within the liver, and also what their behaviour is. Our diagnostic methods for the determination of metastatic liver disease at the moment are extremely imprecise. Physical signs are on the whole helpful, but an enlarged liver in the presence, say, of a tumour of the colon is not necessarily the site of secondary carcinoma. The patient may have two diseases, such as carcinoma of the colon and cirrhosis of the liver. The tests for liver function are not particularly helpful in this problem. A raised alkaline phosphatase in the presence of otherwise normal liver function tests is suggestive of secondary carcinoma, but is by no means diagnostic. Peritoneoscopy will show obvious secondaries, but will not demonstrate secondaries deep within the liver substance. Nor, except at great inconvenience to the patient, can peritoneoscopy be repeated at intervals to study the rate of growth of tumours.

The future here would seem to lie in some sort of scanning process which will determine the presence of tumours and give us a clear idea of their size. Two methods are at present available. One is the possibility of scanning the liver with ultra-sound, which is a technique that is in its infancy but does show that it is capable of considerable development. A more established technique is to give some radio-isotope which is taken up by normal liver tissue and then, by using a special scanner, to determine the areas of the liver which do not take up the radio-isotope. This can give a very clear picture of the size and distribution of metastatic tumours within the liver.

The treatment of liver secondaries is at the present time highly unsatisfactory. Occasionally, when there is only one solitary metastatis, a lobectomy may cure the patient, but these cases are very few and far between.

Intra-arterial chemotherapy, a technique which many of you know about, and has been pioneered in this country by Espiner at the Bristol Royal Infirmary, has been extended by Sullivan, its instigator, to the treatment of liver tumours (Sullivan *et al.*, 1964). At operation he places a catheter permanently in the hepatic artery so that he may deliver chemotherapeutic agents over a very prolonged period of time directly to the liver tumours. In some cases Sullivan has obtained remarkable regression of liver secondaries. This is a technique which is still in its infancy and will surely require a great deal more development if it is to become accepted practice.

There are two other possible approaches to this subject, and one is by immunological methods. The principles of tissue transplantation may be applied to the treatment of malignant disease (Woodruff and Nolan, 1963) (Fig. 2). If the host's immunity to a transplant is temporarily destroyed, either by chemotherapy or by irradiation, then a " graft " of foreign cells can be injected which will either attack the host or the host's tumour.

This technique is also in its infancy, and its further development depends on readily available sources of immunologically competent cells. Dr. Symes in the Department of Surgery here has now found it possible to take human spleen cells and freeze them in liquid nitrogen and store them there, and then bring them back to normal temperatures so that we may use these viable cells as immunologically competent cells in the treatment of malignant disease.

Another possible approach, so far totally unexplored, takes us back once again to the Promethean legend. Reptiles and amphibians are capable of regenerating a tail or a limb after it has been amputated. In these circumstances there is not only proliferation of cells, but the cells are organised to form the structures of the new appendage. This further stage of organisation is due to the liberation of specific organisers in the tissues. In certain circumstances tissue organisers can restore malignant tissue to a normal form. For instance, a chemically induced malignant tumour at the base of a newt's tail may disappear if the newt's tail is then amputated distal to the tumour (Seilern-Aspang and Kratochwil, 1962). When the liver regenerates reorganisation also takes place, and maybe we could turn this process to the patient's advantage in the elimination of malignant tumours from the liver.

Fig. 1.—A general scheme to show the mechanism of hepatic coma. The importance of a portal collateral circulation cannot be too strongly emphasised.
(From Riddell and Jones, 1959).

Fig. 2.—A transplant of a tumour to an isogenic host allows the tumour to continue growing, whereas if a tumour is transplanted to an allogeneic host the cells of the host destroy the tumour.
Allogeneic cells transplanted to an animal with an actively growing tumour may destroy the tumour if the host's defences are depressed.

This concept of using regenerating organising tissue to destroy or reorganise malignant cells you may say is fanciful, but it is only an extension of what we have talked about so far, for the whole of modern liver surgery is dependent on the fact that the liver cells can regenerate, and this power of regeneration is immense and can be set working in the patient's favour.

There are other circumstances in which the liver is so damaged or so destroyed that we see no hope of the regeneration of the patient's own liver, and at this point we have to turn to another great concept of modern biology and this is the transplantation theory.

Replacement of the liver is called for under the following conditions: congenital atresia of the bile ducts, acute liver necrosis, advanced cases of cirrhosis, and when the liver is almost completely destroyed by tumour. Replacement of any organ, whether it be liver or kidney, calls for a phased approach.

The first part of the strategical programme is the ability to keep the patient alive until a suitable graft is available. One of the important factors in promoting rapid advance in renal transplantation has been the availability of the artificial kidney. There seems little hope in the future of developing a mechanical artificial liver, but heart-lung technology has led us to the development of isolated perfusion apparatuses. An isolated liver can be maintained in a normal functioning state on a small specially designed heart-lung machine. Using such a technique it was found that the pig's liver could be satisfactorily perfused with human blood. The next step has logically followed, that the patient can be perfused through a pig's liver and this in fact will support him and perform, over a short period of time, the function of his own liver (Eiseman *et al.*, 1965).

The second problem is to obtain satisfactory grafts. This is always going to be a difficult problem and the liver will need to be obtained rapidly from a person who has recently died. Suitable material is going to be infrequently available, but ideally we should have some method of preserving organs until we need them for transplantation. So far this has proved an insurmountable problem, in that no one has yet succeeded in storing human liver, and keeping it in a state that is suitable for reimplantation after a period of fourteen days. It is likely that the problem is soluble, and certainly by cooling the liver by certain techniques it is possible to preserve in it a large number of the liver cells, so that if you take such a stored liver and test it on an isolated liver perfusion apparatus, it will take up oxygen. At present, however, livers so treated are far too imperfect to be used for transplantation (Moss *et al.*, 1966).

You may well ask at this point, is liver transplantation possible, or is this all a pipe dream? Well, the technique is difficult but not insurmountable, and it has been performed many times in experimental animals with an acceptable mortality. The second part of this problem is, what are the results of transplantation? In the dog these are very encouraging indeed and are in fact much more encouraging than the comparable results for kidney transplantation.

TABLE II

SUMMARY OF PRESENT POSITION: HUMAN LIVER TRANSPLANTS

| Case | Cause of death | Survival, Days |
|---|---|---|
| Starzl 1 | Hæmorrhage | 0 |
| Starzl 2 | Pulmonary emboli | 22 |
| Starzl 3 | Pulmonary emboli | 7½ |
| Starzl 4 | Pulmonary emboli | 6½ |
| Starzl 5 | C.B.D. necrosis | 25 |
| Absolon 1 | C.B.D. necrosis | 13 |

When we turn to apply this knowledge to man, the situation is a gloomy one, in that all humans who so far have received a transplanted liver have died shortly after the operation (Table II). A great deal has been learned from these patients, and it would seem at the present time that more laboratory studies are needed before this technique can be applied with any hope of success to man. The most encouraging part of this is that none of these patients, who have lived for any length of time, have shown any evidence of rejecting the transplanted liver.

Mr. President—I have talked to you for long enough about a subject which fascinates me. It is difficult to sum up what I have to say, but it would appear that modern technological advances and the understanding of biological science does seem to offer to the surgeon some possibilities of extending his usefulness in the treatment of patients with liver disease.

### REFERENCES

Cooke, R. V. and Southwood, W. F. W. (1964). *Brit. J. Surg.,* **51**, 767.

Eiseman, B., Liern, D. S., Raffucci, F. (1965). *Ann. Surg.* **162**, 329.

McDermott, W. V. Jr. and Adams, R. D. (1954). *J. Clin. Invest.,* **33**, 1.

McDermott, W. V. Jr., Greenberger, N. J., Isselbacker, K. J., and Weber, A. L. (1963). *Surgery,* **54**, 56.

Moss, G. S., Reed, P. C., and Riddell, A. G. (1966). *J. Surg. Res.,* **6**, 147.

Riddell, A. G. (1952). *Brit. J. Surg.,* **40**, 251.

Riddell, A. G. and Davies, R. P. (1966). *Proc. Roy. Soc. Med.* (In Press).

Riddell, A. G. and Jones, W. K. (1959). Recent Advances in Surgery, Ed. Taylor, S. London. J. & A. Churchill, Ltd.

Riddell, A. G. and Wilkinson, F. O. W. (1964). *Brit. J. Surg.,* **51**, 669.

Seilern-Aspang, K. and Kratochwil, K. (1962). *J. Embryol. & Exper. Morph.,* **10**, 337.

Starzl, T. E., Marchioro, T. L., Sexton, A. W., Illingworth, B., Waddell, W. R., Faris, T. D., and Herrmann, T. J. (1965). *Surgery,* **57**, 687.

Sullivan, R. D., Norcross, J. W., and Watkins, E. Jr. (1964). *New Eng. J. Med.,* **270**, 321.

Walker, R. M. (1957). *Lancet,* **i**, 57.

Walker, R. M. (1966). *Ann. Roy. Coll. Surg. Eng.,* **38**, 71.

Woodruff, M. F. A., and Nolan, B. (1963). *Lancet,* **ii**, 426.

## Figures and Tables

**Fig. 1. f1:**
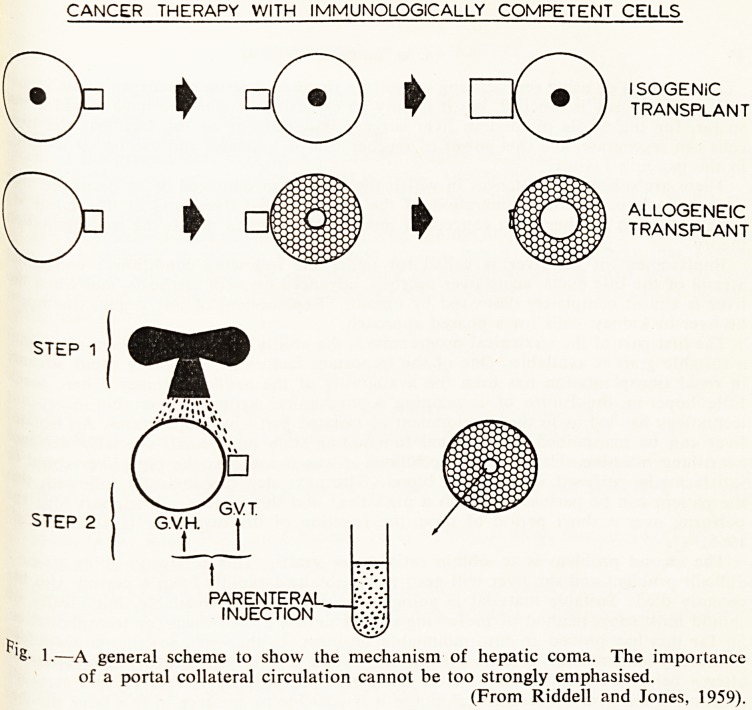


**Fig. 2. f2:**